# Extended blood circulation and joint accumulation of a p(HPMA-*co*-AzMA)-based nanoconjugate in a murine model of rheumatoid arthritis

**DOI:** 10.1186/2052-8426-2-29

**Published:** 2014-09-11

**Authors:** Morten F Ebbesen, Konrad Bienk, Bent W Deleuran, Kenneth A Howard

**Affiliations:** Interdisciplinary Nanoscience Center (iNANO), University of Aarhus, 8000 Aarhus C, Denmark; Department of Molecular Biology and Genetics, University of Aarhus, 8000 Aarhus C, Denmark; Department of Biomedicine, University of Aarhus, 8000 Aarhus C, Denmark

**Keywords:** Arthritis, Collagen Antibody-Induced Arthritis, Extended circulation, HPMA, *in vivo*, Image analysis, Joint accumulation, *N*-(3-azidopropyl)methacrylamide), Poly(*N*-(2-hydroxypropyl)methacrylamide)

## Abstract

**Background:**

We recently synthesized a hydrophilic polymer, poly(*N*-(2-hydroxypropyl)methacrylamide-*co*-*N*-(3-azidopropyl)methacrylamide), p(HPMA-*co*-AzMA), by RAFT polymerization using a novel azide-containing methacrylamide monomer that through a post modification strategy using click chemistry enabled facile preparation of a panel of versatile and well-defined bioconjugates. In this work we screen a panel of different molecular weight (Mw) fluorescently tagged p(HPMA-*co*-AzMA) in healthy mice, by live bioimaging, to select an extended circulatory half-life material for investigating joint accumulation in a murine collagen antibody-induced arthritis model.

**Findings:**

Fluorescence image analysis revealed half-lifes of <20 min, 2.8 h and 6.4 h for p(HPMA-*co*-AzMA) of 15, 36 and 54 kDa, respectively, with ~10% polymer retained in the blood after 24 h for the highest Mw. p(HPMA-*co*-AzMA) of 54 kDa showed enhanced accumulation in the joints of the arthritic mouse model with a bioavailability (AUC = 1783% · h) ~12 times higher (*P* = 0.01) than healthy control (AUC = 148% · h).

**Conclusions:**

p(HPMA-*co*-AzMA) of 54 kDa exhibited extended circulatory half-life and preferential accumulation in inflamed joints of a murine model of rheumatoid arthritis (RA). This combined with well-defined polymer size and versatility for conjugation of a range of biomolecules promotes p(HPMA-*co*-AzMA) for potential applications in the delivery of drugs for treatment of RA.

**Electronic supplementary material:**

The online version of this article (doi:10.1186/2052-8426-2-29) contains supplementary material, which is available to authorized users.

## Findings

### Introduction

Rheumatoid arthritis (RA) is a chronic progressive autoimmune disease affecting ~ 1% of the population causing cartilage and bone destruction in synovial joints [1]. The pathogenesis involves synovial infiltration by circulatory immune cells that induce inflammation, modulated predominantly by proinflammatory cytokines such as tumor necrosis factor alfa (TNFα) [2, 3]. Common clinical treatments include nonsteroidal anti-inflammatory drugs (NSAIDs), glucocorticoids, and disease-modifying antirheumatic drugs (DMARDs) such as anti-TNFα antibodies. Although effective in many patients, these therapeutics have associated side-effects such as myelosuppression and increased infection risk, primarily due to a general systemic immune suppression that necessitates alternative approaches [1, 4]. RA associated angiogenesis, necessary for formation of pannus and invasion of inflammatory cells into the synovial tissue, has been identified to establish a macromolecular retention effect [5–7], conceptually similar to the EPR effect for tumors [8]. Hydrophilic polymers synthesized with high molecular weights (Mw) enable the construction of polymer macromolecular drugs [9] with extended blood circulation and specific targeting to arthritic joints. This has been used for local suppression of proinflammatory cytokines such as TNFα [10] that may increase the clinical efficacy and reduce generalized side effects.

**Figure 1:**
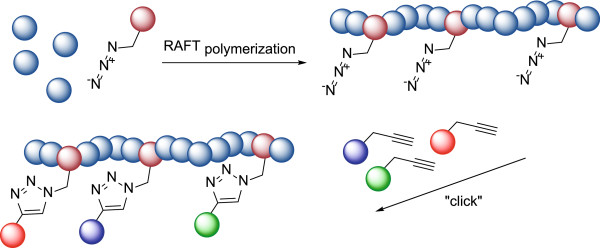
**Synthesis and functionalization of the p(HPMA-** ***co*** **-AzMA) copolymer as a versatile method for production of polymer bioconjugates (Reproduced from ref. 13 with permission from John Wiley and Sons).**

The field of polymer therapeutics requires increased attention to defining and minimizing macromolecule heterogeneity and polydispersity due to its great influence on pharmacokinetics, safety and efficacy of polymer therapeutics and to identify precise polymer characteristics required for delivery e.g. to RA affected tissue [11, 12]. We have recently synthesized a novel azide containing copolymer, poly(*N*-(2-hydroxypropyl)methacrylamide-*co*-*N*-(3-azidopropyl)methacrylamide), (p(HPMA-*co*-AzMA)), through a versatile post-modification procedure to produce a panel of well-defined polymer bioconjugates of narrow PdI (Figure 1) [13]. Compared to existing bioconjugation strategies for RA [14, 15], this may provide superior polymer characteristics in terms of flexibility for modification and low polydispersity.

### Research hypothesis

We hypothesize that the p(HPMA-*co*-AzMA) constructs will exhibit prolonged blood circulation and specific accumulation in inflamed joints in a murine model of rheumatoid arthritis supporting its potential therapeutic application for RA.

### Methods

For blood circulation studies, a PBS solution (100 μL) of p(HPMA-*co*-AzMA) polymers (4 mg/kg) of different Mw (15, 36 and 54 kDa) labeled with an Atto680 dye (ATTO-TEC GmbH, Siegen, Germany), or PBS only control was administered intravenously (i.v.) in healthy BALB/c male mice (8 week-old, Taconic Europe, Ry, Denmark). Blood volumes (~80 μL, max 2 pr. animal) were sampled from 5 min up to 24 h post-injection and the blood plasma was subsequently transferred to narrow 20 uL glass capillary tubes (minicaps, Hirschmann) and imaged with the IVIS® Spectrum using a 675/720 nm filterset and analyzed using the Living Image software version 4.3 (PerkinElmer).

Nine week-old, male, DBA/1 (Taconic Europe, Ry, Denmark) were used for the collagen antibody-induced arthritis (CAIA) model [16] with intraperitoneal (i.p.) injection of the monoclonal antibody mix (Arthrogen-CIA® arthritogenic monoclonal antibody 5 clones cocktail kit, Chondrex, Inc. Redmond, USA) and a subsequent LPS injection 3 days later. The severity of the arthritic condition was monitored by daily clinical scoring of joints (1–4) [16] with symptoms developing ~ day 3 and peaking around day 8. Healthy control mice (DBA/1) were injected i.p. with PBS.

For the joint accumulation studies, a PBS solution of the 54 kDa Atto680-labeled (0.57 w/w%) p(HPMA-*co*-AzMA), or the free dye, was administered by intravenous (i.v.) injection to arthritic and control animals at a polymer dose of 4 mg/kg or the dye-equivalent amount (22.6 ug/kg) of free dye. This dose was comparable to existing studies [17, 18] and at a concentration by which the polymer conjugated dye could be easily visualized using *in vivo* fluorescence imaging. Joint fluorescence was monitored before, and up to, 24 h post-injection by IVIS Spectrum *in vivo* imaging of isoflurane (2.5%) anesthetized mice. Multispectral fluorescence images covering the absorption-emission profile of the Atto680 dye were acquired using the Living Image software (PerkinElmer). Images were spectrally unmixed to subtract background fluorescence, which enabled the total fluorescence emission from joint areas from each mouse to be quantified. Measured intensities of the fluorescence emission are generally linear dependent on fluorophore concentration, extinction coefficient and quantum yield, which enables a direct correlation to polymer levels in the blood or tissue [19].

All image data was analyzed using Prism (GraphPad Software Inc.). Blood polymer levels were normalized to the value measured at 5 min and joint polymer levels to the highest mean value and presented as % with SEM. Students t-test was performed to determine data significance.

All procedures of animal work were performed according to international recognized guidelines and the animal experimental protocols approved by ‘The Experimental Animal Inspectorate in Denmark’ under The Danish Veterinary and Food Administration, Ministry of Food, Agriculture and Fisheries (Registration number: 2013 - 15 - 2934 - 00789-C2, issue date: March 5, 2013).

### Results and discussion

**Figure 2:**
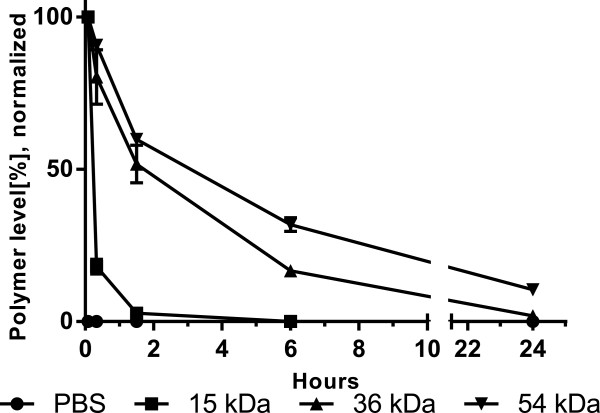
**Blood circulation profiles. Blood circulation profiles of dye-labeled p(HPMA-** ***co*** **-AzMA) of 15, 36 and 54 kDa (PBS as control) in BALB/c mice.** Polymer levels were normalized to the value measured at 5 min. 10% of the 54 kDa polymer was retained in the blood at 24 h Polymer bioavailability (AUC) are 698, 313 and 45% · h for polymers of 54, 36 and 15 kDa, respectively. Polymer circulation half-life was calculated to 6.4, 2.8 and < 0.33 h for polymers of 54, 36 and 15 kDa, respectively. Averages of n = 3 mice are shown with SEM.

Blood circulation profiles (Figure 2) constructed from blood plasma fluorescent polymer levels showed a Mw-dependent prolonged blood retention of the copolymers. At 24 h, the 54 kDa copolymer was still retained in the blood at ~10% and accordingly displayed a significant (*P* < 0.03) higher bioavailability (area under the curve, AUC = 698% · h) compared to 36 kDa (AUC = 313% · h) and 15 kDa (AUC = 45% · h) polymers [see Additional file 1: Figure S1]. First order clearance kinetics were modeled from 1 h post i.v. injection of dye-labeled copolymers of different Mw and exhibited a half-life of < 20 min for polymers of 15 kDa that increased up to 2.8 h for 36 kDa and 6.4 h for 54 kDa polymers, in general accordance with previous studies on HPMA copolymers [18]. Renal filtration commonly occurs for polymers with a hydrodynamic radius below 5 nm that corresponds to ~30 to 50 kDa Mw, however, with renal clearance of linear polymers, such as in this work, being up to 10 times higher than those of more globular-shape [20]. In this work, the highest Mw p(HPMA-*co*-AzMA) did not seem to be excessively larger than the renal clearance limit, but still exhibiting prolonged circulation, and was, thus, selected for evaluation in arthritic mice.

**Figure 3:**
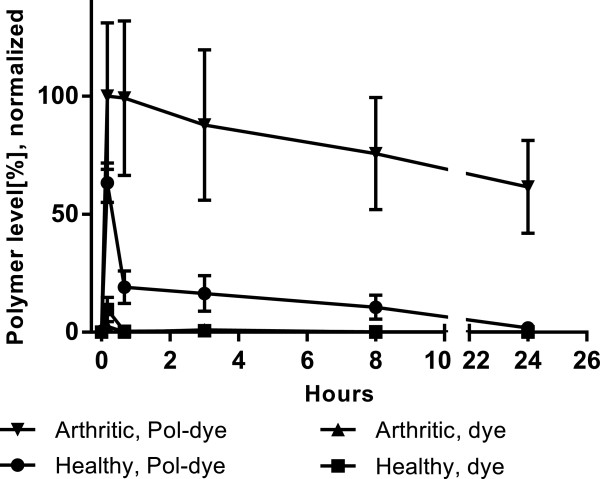
**Dye-labeled p(HPMA-** ***co*** **-AzMA) joint accumulation profiles.** 62% of the 54 kDa dye-labeled copolymer (Pol-dye) was retained in arthritic joints of DBA/1 mice at 24 h. Polymer bioavailability (AUC) was 1783 and 148% · h in arthritic (n = 16 joints, 4 mice) and healthy (n = 16 joints, 4 mice) joints. Dye alone was included as control with AUC of 4.48 and 8.96% · h in arthritic (n = 4 joints, 1 mouse) and healthy (n = 8 joints, 2 mice). Joint polymer levels are normalized to the highest mean value and averages shown with SEM.

**Figure 4:**
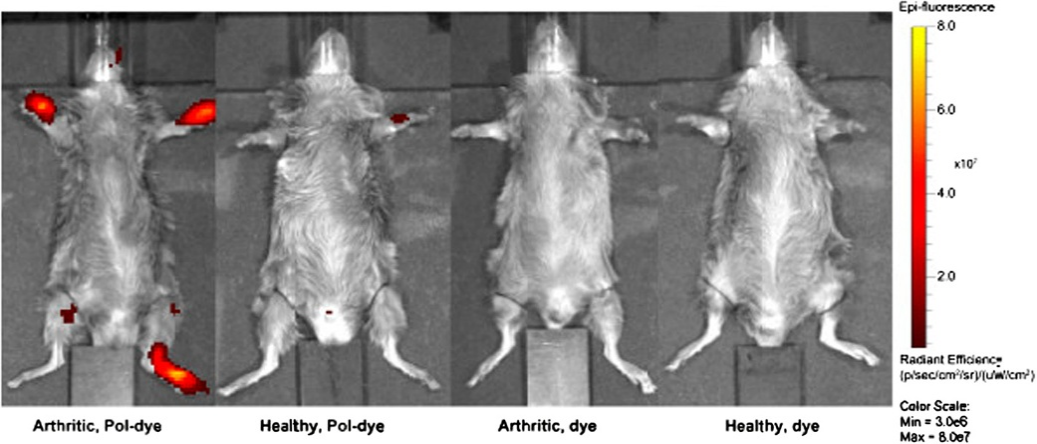
**Paw fluorescence from arthritic and non-arthritic mice 24 h post-injection.** IVIS Spectrum *in vivo* imaging of arthritic and non-arthritic mice 24 h post-injection of a 54 kDa dye-labeled copolymer (Pol-dye) and dye alone. Multispectral fluorescence images were unmixed to subtract background fluorescence enabling visualization of the dye and dye-labeled polymers. The images show representative examples of polymer accumulation in arthritic joints, minute amounts of polymer in joints of healthy mice, and no presence of dye alone as control that correspond to Figure 3.

Enhanced passive accumulation of the 54 kDa dye-grafted p(HPMA-*co*-AzMA) in the joints of arthritic mice compared to healthy was demonstrated (Figure 3 and Figure 4) with the bioavailability in arthritic tissue (AUC = 1783% · h) being ~12 times higher (*P* = 0.01) compared to healthy tissue (AUC = 148% · h) (see Additional file 2: Figure S2). Additionally, at 24 h, polymer levels in the blood of arthritic animals were lower than that of healthy (see Additional file 3: Figure S3), but showed a higher level in arthritic paws compared to healthy (Figure 3 and Figure 4). This suggests the polymer to be escaping blood circulation faster in the arthritic mice, probably due to accumulation in the arthritic joints. The polymer levels in healthy mice joints initially increased by 63% within 10 minutes but subsequently quickly declined, probably as a result of blood clearance of the polymer in general and a healthy microvasculature not exhibiting permeability and polymer extravasation effects. Free dye in arthritic or non-arthritic tissue was rapidly excreted and almost cleared before the first time point at 10 min (Figure 3, 10 min).

**Figure 5:**
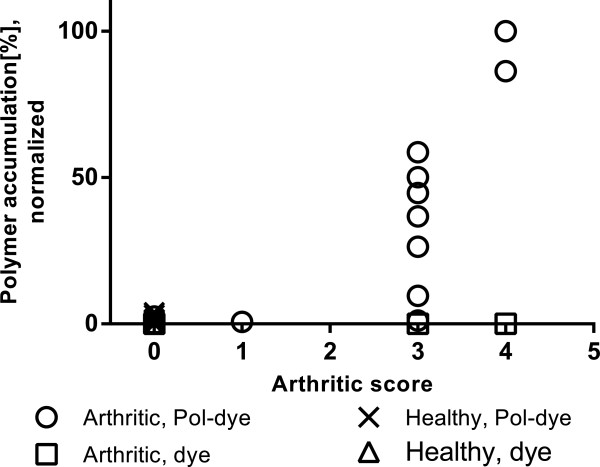
**Correlation plot of arthritic score against polymer accumulation in DBA/1 mice joints at 24 h.** Spearman correlation coefficient, r = 0.89 with *P* < 0.0001 supports a direct correlation.

The arthritic scores, as an indicator of inflamed severity, were not entirely uniform with individual mice exhibiting combinations of joint scores (1–4). A Spearman test of polymer accumulation (at 24 h) and arthritic score correlation was, therefore, performed (Figure 5), supporting strong correlation between polymer accumulation and the arthritic condition (r = 0.89, *P* < 0.0001).

In summary, this work demonstrates the *in vivo* characteristics of a new azide containing copolymer, (p(HPMA-*co*-AzMA)), which offers a more versatile and well-defined alternative to existing bioconjugate systems. Attractive *in vivo* properties such as prolonged blood retention and specific accumulation in inflamed joints of RA illustrate the potential of the material for systemic delivery of anti-inflammatory drugs for local effects in the treatment of RA.

## Electronic supplementary material

Additional file 1: Figure S1: Blood plasma bioavailability (AUC), in healthy BALB/c mice, of p(HPMA-*co*-AzMA) of 15, 36 and 54 kDa calculated from blood plasma polymer profiles in Figure 2. (PDF 103 KB)

Additional file 2: Figure S2: Bioavailability (AUC) of dye-labeled p(HPMA-*co*-AzMA) of 54 kDa and free dye in arthritic and healthy joints. AUC’s are calculated from p(HPMA-*co*-AzMA) joint accumulation profiles in Figure 3. (PDF 106 KB)

Additional file 3: Figure S3: Blood plasma levels of dye-labeled p(HPMA-*co*-AzMA) and free dye at 24 h. Polymer levels in the blood indicate that the polymer escapes blood circulation faster in the arthritic mice possible due to accumulation in the arthritic mice joints. Levels are normalized to the highest mean value and averages shown with SEM. (PDF 92 KB)
