# Supplementary figures and images for: Extended blood circulation and joint accumulation of a p(HPMA-co-AzMA)-based nanoconjugate in a murine model of rheumatoid arthritis
Source: Mol Cell Ther. 2014 Sep 11;2:29. doi: 10.1186/2052-8426-2-29 (PMC4452064; doi:10.1186/2052-8426-2-29)

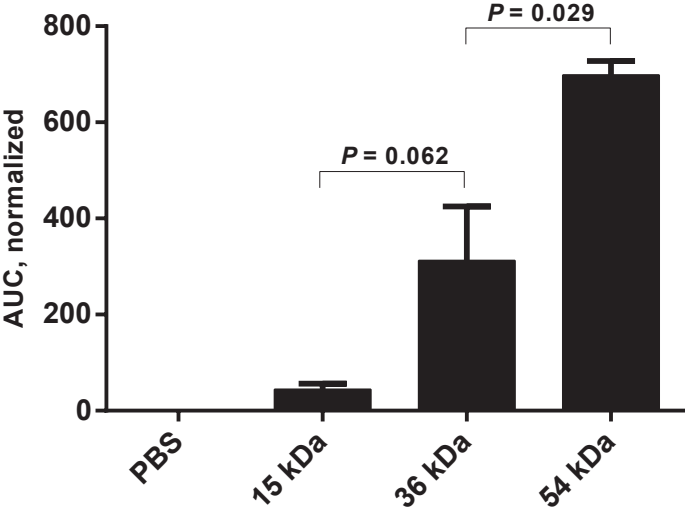

Supplement: Supplementary file 1 — Additional file 1: Figure S1: Blood plasma bioavailability (AUC), in healthy BALB/c mice, of p(HPMA-co-AzMA) of 15, 36 and 54 kDa calculated from blood plasma polymer profiles in Figure 2. (PDF 103 KB) [file 40591_2014_33_MOESM1_ESM.pdf]

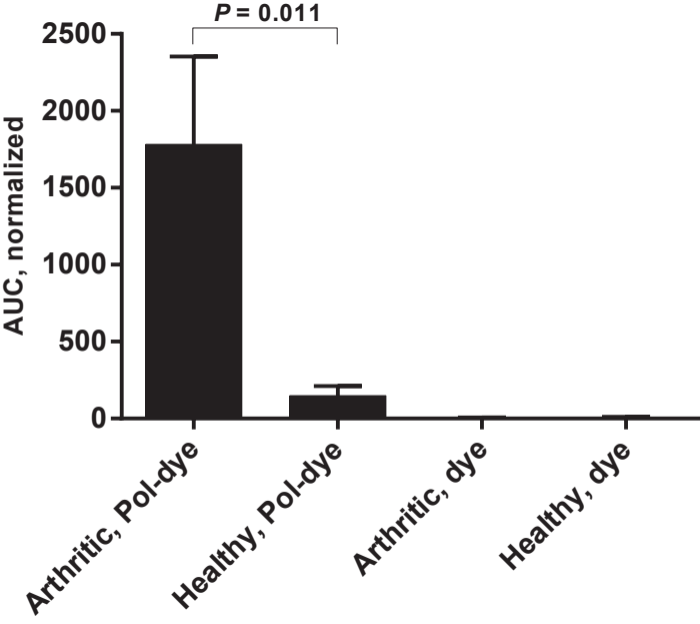

Supplement: Supplementary file 2 — Additional file 2: Figure S2: Bioavailability (AUC) of dye-labeled p(HPMA-co-AzMA) of 54 kDa and free dye in arthritic and healthy joints. AUC’s are calculated from p(HPMA-co-AzMA) joint accumulation profiles in Figure 3. (PDF 106 KB) [file 40591_2014_33_MOESM2_ESM.pdf]

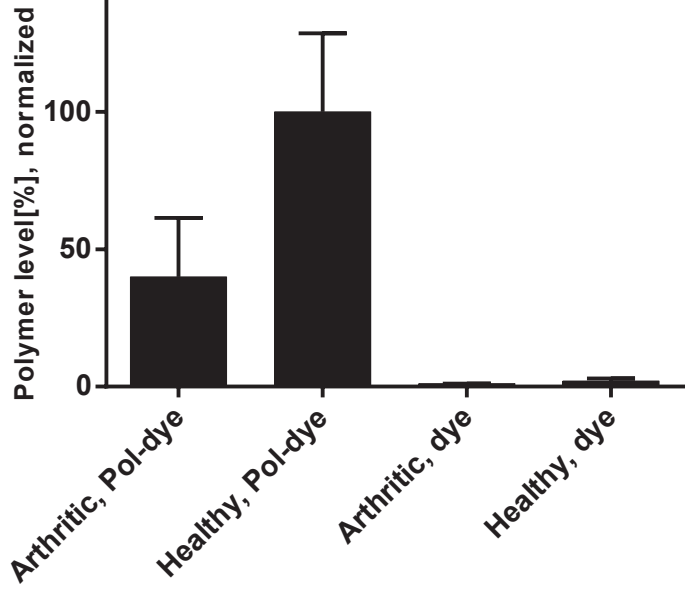

Supplement: Supplementary file 3 — Additional file 3: Figure S3: Blood plasma levels of dye-labeled p(HPMA-co-AzMA) and free dye at 24 h. Polymer levels in the blood indicate that the polymer escapes blood circulation faster in the arthritic mice possible due to accumulation in the arthritic mice joints. Levels are normalized to the highest mean value and averages shown with SEM. (PDF 92 KB) [file 40591_2014_33_MOESM3_ESM.pdf]
